# Copenhagen index (CPH-I) is more favorable than CA125, HE4, and risk of ovarian malignancy algorithm (ROMA): Nomogram prediction models with clinical-ultrasonographic feature for diagnosing ovarian neoplasms

**DOI:** 10.3389/fsurg.2022.1068492

**Published:** 2023-01-13

**Authors:** Zixuan Song, Xiaoxue Wang, Jiajun Fu, Pengyuan Wang, Xueting Chen, Dandan Zhang

**Affiliations:** ^1^ Department of Obstetrics and Gynecology, Shengjing Hospital of China Medical University, Shenyang, China; ^2^ Department of Health Management, Shengjing Hospital of China Medical University, Shenyang, China; ^3^ Department of Pathology, Shengjing Hospital of China Medical University, Shenyang, China; ^4^ Department of Radiology, Shengjing Hospital of China Medical University, Shenyang, China

**Keywords:** ovarian neoplasms, epithelial ovarian cancer, CA125, HE4, risk of ovarian malignancy algorithm (ROMA), Copenhagen index (CPH-I), nomogram, prediction model

## Abstract

**Background:**

We aimed to analyze the benign and malignant identification efficiency of CA125, HE4, risk of ovarian malignancy algorithm (ROMA), Copenhagen Index (CPH-I) in ovarian neoplasms and establish a nomogram to improve the preoperative evaluation value of ovarian neoplasms.

**Methods:**

A total of 3,042 patients with ovarian neoplasms were retrospectively classified according to postoperative pathological diagnosis [benign, *n* = 2389; epithelial ovarian cancer (EOC), *n* = 653]. The patients were randomly divided into training and test cohorts at a ratio of 7:3. Using CA125, HE4, ROMA, and CPH-I, Receiver operating characteristic (ROC) curves corresponding to different truncation values were calculated and compared, and optimal truncation values were selected. Clinical and imaging risk factors were calculated using univariate regression, and significant variables were selected for multivariate regression analysis combined with ROMA and CPH-I. Nomograms were constructed to predict the occurrence of EOC, and the accuracy was assessed by external validation.

**Results:**

When the cutoff value of CA125, HE4, ROMA, and CPH-I was 100 U/ml, 70 pmol/L, 12.5/14.4% (premenopausal/postmenopausal) and 5%, respectively, the AUC was 0.674, 0.721, 0.750 and 0.769, respectively. From univariate regression, the clinical risk factors were older age, menopausal status, higher birth rate, hypertension, and diabetes; imaging risk factors were multilocular tumors, solid nodules, bilateral tumors, larger tumor diameter, and ascites. The AUC of the nomogram containing ROMA and CPH-I was 0.8914 and 0.9114, respectively, which was better than the prediction accuracies of CA125, HE4, ROMA, and CPH-I alone. The nomogram with CPH-I was significantly better than that with ROMA (*P* < 0.001), and a nomogram decision curve analysis (DCA) containing CPH-I seemed to have better clinical benefits than ROMA. For external validation of this nomogram containing ROMA and CPH-I, the C-indices were 0.889 and 0.900, and the calibration curves were close to 45°, showing good agreement with the predicted values.

**Conclusion:**

We conclude that CPH-I and ROMA have higher diagnostic values in the preoperative diagnosis of EOC than other single tumor markers like CA125 or HE4. A nomogram based on CPH-I and ROMA with clinical and ultrasonic indicators had a better diagnostic value, and the CPH-I nomogram had the highest diagnostic efficacy.

## Background

The onset of ovarian cancer (OC) is insidious, and early diagnosis is difficult. Its mortality rate is the fifth highest among all cancers and ranks first among gynecological cancers (1). The 5-year survival rate of ovarian cancer is less than 30% (2), among which epithelial ovarian cancer (EOC) accounts for nearly 90% of all malignant ovarian diseases and is one of the fifth leading causes of cancer death in women, with an overall 5-year survival rate of about 46% (3, 4). Due to the lack of typical clinical symptoms and effective screening methods in the early stage of EOC (stage I and stage II), 70%–75% of patients are diagnosed at an advanced stage, and the 5-year survival rate of these patients is only 20%–30% (5). Although the 5-year survival rate for early-stage OC is high, as high as 90% to 95% in FIGO stage I patients, only 15% of patients are found by chance during visits for other illnesses or physical examinations (6). Delayed diagnosis of EOC results in difficult treatment and a poor prognosis.

Currently, the most commonly used serum tumor marker for EOC diagnosis is cancer antigen 125 (CA125) (7); however, CA125 has limited diagnostic specificity and low overall predictive value (8). For early OC, some studies reported the results of sensitivity studies as low as 25% (25%–75%) in stage I and 61% (61%–96%) in stage II (9). In addition, CA125 is elevated to a certain extent in some non-malignant gynecological diseases, non-gynecological cancers, or physiological conditions such as pregnancy and menstruation (10), which results in low specificity and limits its clinical application.

Human epididymis protein 4 (HE4), a novel tumor marker, has been approved for diagnosing OC because of its similar sensitivity and higher specificity to CA125 (11). Some studies have shown that HE4 is expressed at low levels in normal ovarian tissue but is more commonly expressed in malignant ovarian tumors, especially serous adenocarcinoma, while there is no significant increase in benign ovarian lesions (12–14). In serous OC and endometrioid adenocarcinoma, 93% and 100% of cases, respectively, had elevated HE4 (15). Therefore, in 2008, the US Food and Drug Administration (FDA) approved HE4 for monitoring patients with EOC for disease recurrence or progression (16). HE4 levels were also reported as a marker for recurrence in patients with a normal CA125 at initial diagnosis (17, 18). However, HE4 is influenced by menopausal status and age (19). In addition, the HE4 threshold for different devices or different methods to distinguish benign from malignant ovarian masses is controversial (20).

In 2009, Moore first proposed the risk of ovarian malignancy algorithm (ROMA) model by combining CA125, HE4, and menopausal status (21). In 2011, ROMA was approved by the FDA for the risk assessment of ovarian disease because of its increased sensitivity, specificity, and accuracy in OC prediction. Thus, evaluating ovarian neoplasms can guide patients with suspected OC to receive standardized treatment as soon as possible. Several studies have confirmed ROMA's clinical usefulness of the ROMA (22, 23). Menopause cannot be accurately defined because it is subject to variables like age, time of menopause, serum follicle-stimulating hormone (FSH) level, and race (24). Compared with menopause, age is a more objective indicator. Based on this, Karlsen proposed the Copenhagen Index (CPH-I) in 2015, a model combining CA125, HE4, and patient age, with a higher area under the curve (AUC) than ROMA (25). This model has been validated in several international studies to differentiate EOC from benign tumors (26, 27). In addition, Yoshida et al. showed that the inclusion of non-epithelial ovarian cancer and borderline tumors and CHP-I also had effects (28). Age is readily available compared to the ROMA criteria for menopausal status and can significantly improve the accuracy of the index. In addition, the CPH-I uses the same formula for premenopausal and postmenopausal women, making the assessment more practical and concise.

Because CPH-I is a relatively new model proposed in recent years, there are few related studies. A nomogram is a visual representation of an individual's (positive) probability of an outcome based on a regression model. Its basic principle is to determine the scoring standard according to the size of the independent variable regression coefficient in the prediction model, assign each value level of the predictor a score, and then calculate the individual's total score. Finally, the probability of individual outcome events was calculated using the conversion function between the total score and the probability of the outcome (29). This study aimed to evaluate the clinical significance of CA125, HE4, ROMA, and CPH-I in differentiating benign and malignant ovarian neoplasms using an evidence-based approach. We also aimed to establish a nomogram to provide an EOC evaluation scale by integrating different prediction methods and other clinical factors and provide a reliable nomogram score simulation decision for clinicians to conduct standardized clinical practice.

## Methods

### Patients

**Figure 1:**
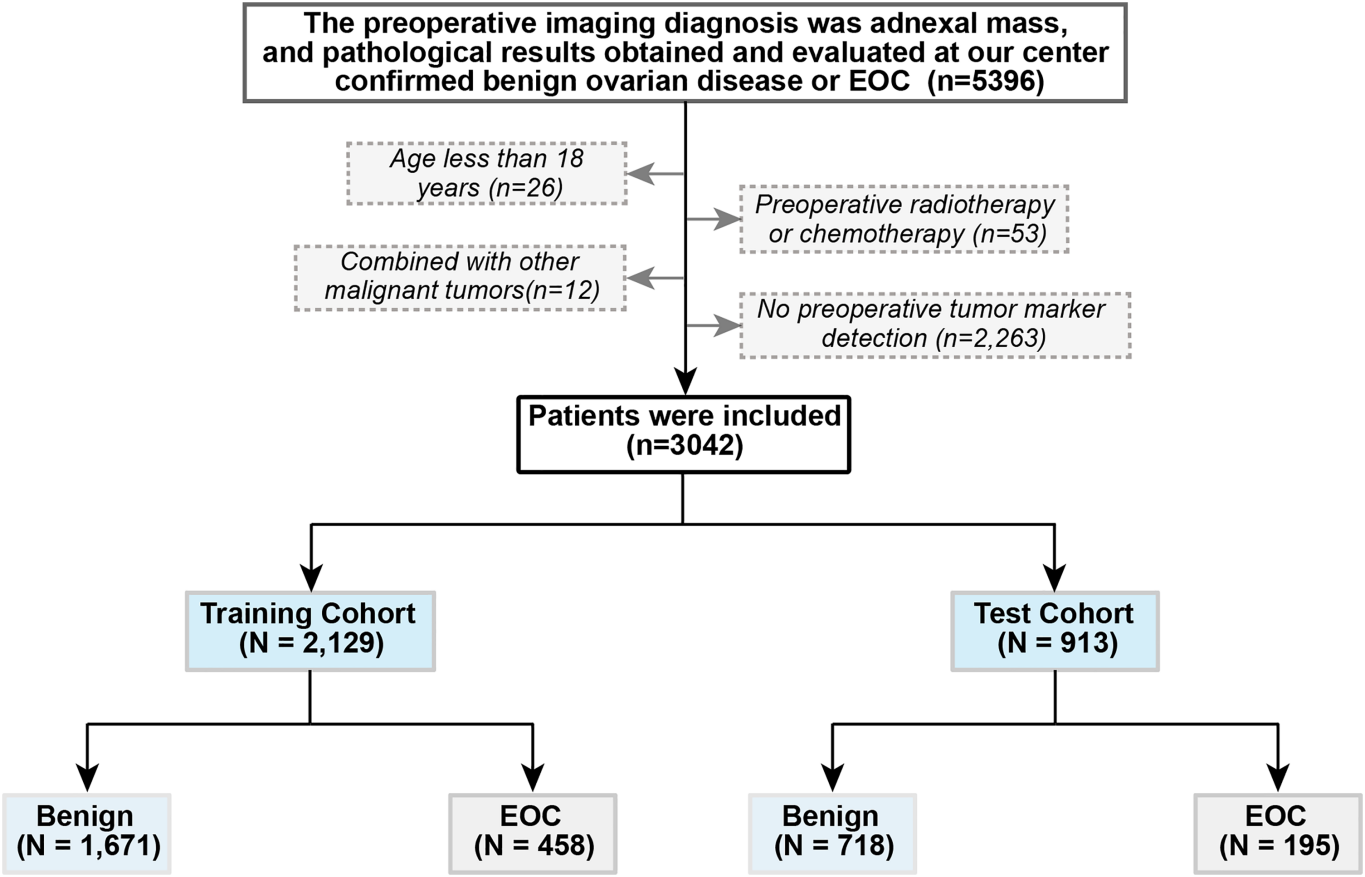
Flowchart of patient selection. EOC, epithelial ovarian cancer.

This observational retrospective study included women diagnosed with ovarian cysts or pelvic masses who visited the Shengjing Hospital of China Medical University between January 2017 and December 2021. The inclusion criteria were as follows: (1) pre-operation ovarian cysts or pelvic masses found on pelvic imaging [ultrasound, computed tomography scan, or magnetic resonance imaging (MRI)]; and (2) pathological results obtained and evaluated at our center confirmed benign ovarian disease or EOC. The exclusion criteria were as follows: (1) age <18 years, (2) preoperative radiotherapy and chemotherapy, (3) combined with other malignant tumors, and (4) no preoperative tumor marker detection. The patients were randomly divided into training and test cohorts at a ratio of 7:3. The patient selection criteria flowchart is shown in Figure 1. This study was approved by the Ethics Committee of the Shengjing Hospital of China Medical University (ethics no. 2022PS134K). Written informed consent was obtained prior to completion of the MRI exams.

### Data collection

Clinical data of the patients included in this study were collected, including age, menopausal status, parity, hypertension, and diabetes. Preoperative imaging results were collected, including whether the pelvic masses were multilocular tumors, contained solid components, bilateral tumors, ascites, and largest diameter. Preoperative tumor marker information, including CA125 and HE4 levels, was collected. Serum CA125 and HE4 concentrations were detected using the automatic electrochemiluminescence immunoassay system at our center (Roche Cobas E601) and its supporting reagents. Based on this information, ROMA (21) was computed using the following formula:

$$\begin{array}{rl} Premenopausal\; & :\;Predictive\;Index\;(PI)\\ & :\;-12.0+2.38*LN\;(HE4)\\ & \;\;\;\;+0.0626*LN\;(CA125)\\ Postmenopausal\; & :\;Predictive\;Index\;(PI)\\ & :\;-8.09+1.04*LN\;(HE4)\\ & \;\;\;\;+0.732*LN\;(CA125)\\ Predicted\;Probability\;(PP) & =exp\;(PI)/[1/exp\;(PI)]*100\% \end{array}$$

CPH-I was computed according to the following calculation formula (25):

$$\begin{array}{rl} \mathrm{CPH}-I= & -14.0647+1.0649*\mathrm{lo}g_{2}(\mathrm{HE}4)+0.6050\\ & *\mathrm{lo}g_{2}(\mathrm{CA}125)+0.2672*\mathrm{age}/10\\ \mathrm{PP}= & \;\;e^{\mathrm{CPH}-I}\;/[1+e^{\mathrm{CPH}-I}\;]*100\% \end{array}$$

The endpoints of this study were postoperative pathological findings, which were classified as benign or EOC.

Receiver operating characteristic (ROC) curves corresponding to different cutoff values for tumor markers were compared, and the optimal cutoff values for ROMA and CPH-I were selected. Different cutoff values for CA125, HE4, ROMA, and CPH-I have been reported in the previous literature (21, 25, 28, 30, 31). ROC curves were plotted from these cutoff values, and the AUC was compared. The closer the AUC is to 1.0, the higher the diagnostic performance (32). The cutoff values of ROMA and CPH-I corresponding to the highest AUC were obtained for subsequent analyses.

### Data analysis

All data were analyzed in the RStudio environment using R Version 3.6.3 (R Foundation for Statistical Computing, Vienna, Austria, http://www.r-project.org). Univariate and multivariate logistic regression analyses of the clinical data, imaging indicators, and tumor indicators were performed to evaluate the risk factors associated with EOC. ROC analysis was used to divide patients into high-risk and low-risk groups based on cut-off values. Odds ratios and 95% confidence intervals were also calculated. Statistical significance was set at *P* < 0.05. Nomograms were constructed to predict EOC occurrence based on the related risk factors using multivariate logistic analyses. The prediction ability of the different prediction models was evaluated based on the AUC with good recognition ability. The clinical effect of the nomogram was evaluated by decision curve analysis (DCA) and net benefit at each risk threshold probability (33).

## Results

### Patients' characteristics

From January 2017 to December 2021, 5,396 patients underwent surgical treatment in our hospital for benign ovarian disease or EOC diagnosed by pathology. Based on the exclusion criteria, 3,042 cases were included. Among them, 2,389 were benign, and 653 were EOC. The patients were divided into a training cohort (*n* = 2129) and a test cohort (*n* = 913). Specific patient characteristics are shown in Table 1.

**Table 1:** Patients’ characteristics.

Variables	Training Cohort (*N* = 2,129)		Test Cohort (*N* = 913)		Total (*N* = 3,042)	
	Benign (*N* = 1,671)	EOC (*N* = 458)	Benign (*N* = 718)	EOC (*N* = 195)	Benign (*N* = 2,389)	EOC (*N* = 6,53)
**Age (years)**						
<40	1,020 (61%)	96 (21%)	432 (60%)	45 (23%)	1,452 (61%)	141 (22%)
40–59	524 (31%)	242 (53%)	230 (32%)	106 (54%)	754 (32%)	348 (53%)
≥60	127 (7.6%)	120 (26%)	56 (7.8%)	44 (23%)	183 (7.7%)	164 (25%)
**Postmenopausal**						
No	1,321 (79%)	186 (41%)	559 (78%)	89 (46%)	1,880 (79%)	275 (42%)
Yes	350 (21%)	272 (59%)	159 (22%)	106 (54%)	509 (21%)	378 (58%)
**Parity**						
0	479 (29%)	46 (10%)	207 (29%)	19 (9.7%)	686 (29%)	65 (10.0%)
1	1,069 (64%)	371 (81%)	452 (63%)	163 (84%)	1,521 (64%)	534 (82%)
≥2	123 (7.4%)	41 (9.0%)	59 (8.2%)	13 (6.7%)	182 (7.6%)	54 (8.3%)
**Hypertension**						
No	1,468 (88%)	328 (72%)	614 (86%)	144 (74%)	2,082 (87%)	472 (72%)
Yes	203 (12%)	130 (28%)	104 (14%)	51 (26%)	307 (13%)	181 (28%)
**Diabetes**						
No	1,508 (90%)	379 (83%)	651 (91%)	149 (76%)	2,159 (90%)	528 (81%)
Yes	163 (9.8%)	79 (17%)	67 (9.3%)	46 (24%)	230 (9.6%)	125 (19%)
**Multilocular tumor**						
No	1,208 (72%)	153 (33%)	517 (72%)	71 (36%)	1,725 (72%)	224 (34%)
Yes	463 (28%)	305 (67%)	201 (28%)	124 (64%)	664 (28%)	429 (66%)
**Contains solid components**						
No	1,373 (82%)	172 (38%)	592 (82%)	63 (32%)	1,965 (82%)	235 (36%)
Yes	298 (18%)	286 (62%)	126 (18%)	132 (68%)	424 (18%)	418 (64%)
**Bilateral tumor**						
No	1,484 (89%)	350 (76%)	630 (88%)	143 (73%)	2,114 (88%)	493 (75%)
Yes	187 (11%)	108 (24%)	88 (12%)	52 (27%)	275 (12%)	160 (25%)
**Largest diameter (cm)**						
<5	383 (23%)	49 (11%)	165 (23%)	24 (12%)	548 (23%)	73 (11%)
5–14	1,087 (65%)	238 (52%)	481 (67%)	104 (53%)	1,568 (66%)	342 (52%)
≥15	201 (12%)	171 (37%)	72 (10%)	67 (34%)	273 (11%)	238 (36%)
**Ascites**						
No	1,579 (94%)	324 (71%)	658 (92%)	142 (73%)	2,237 (94%)	466 (71%)
Yes	92 (5.5%)	134 (29%)	60 (8.4%)	53 (27%)	152 (6.4%)	187 (29%)
CA125 (U/mL)	23 (14, 53)	97 (24, 426)	22 (14, 48)	121 (25, 645)	23 (14, 51)	100 (24, 459)
**CA125 (cutoff: 100 U/ml)**						
Low	1,471 (88%)	244 (53%)	652 (91%)	103 (53%)	2,123 (89%)	347 (53%)
High	200 (12%)	214 (47%)	66 (9.2%)	92 (47%)	266 (11%)	306 (47%)
**CA125 (cutoff: 35/65 U/ml** ^a^ **)**						
Low	1,110 (66%)	187 (41%)	489 (68%)	80 (41%)	1,599 (67%)	267 (41%)
High	561 (34%)	271 (59%)	229 (32%)	115 (59%)	790 (33%)	386 (59%)
**CA125 (cutoff: 35/100 U/ml** ^a^ **)**						
Low	1,123 (67%)	204 (45%)	492 (69%)	86 (44%)	1,615 (68%)	290 (44%)
High	548 (33%)	254 (55%)	226 (31%)	109 (56%)	774 (32%)	363 (56%)
HE4 (pmol/L)	46 (40, 55)	79 (52, 256)	46 (39, 54)	73 (48, 256)	46 (40, 54)	79 (51, 256)
**HE4 (cutoff: 70 pmol/L)**						
Low	1,552 (93%)	223 (49%)	678 (94%)	103 (53%)	2,230 (93%)	326 (50%)
High	119 (7.1%)	235 (51%)	40 (5.6%)	92 (47%)	159 (6.7%)	327 (50%)
**HE4 (cutoff: 120 pmol/L)**						
Low	1,651 (99%)	306 (67%)	711 (99%)	128 (66%)	2,362 (99%)	434 (66%)
High	20 (1.2%)	152 (33%)	7 (1.0%)	67 (34%)	27 (1.1%)	219 (34%)
**HE4 (cutoff: 70/140 pmol/L** ^a^ **)**						
Low	1,611 (96%)	294 (64%)	702 (98%)	120 (62%)	2,313 (97%)	414 (63%)
High	60 (3.6%)	164 (36%)	16 (2.2%)	75 (38%)	76 (3.2%)	239 (37%)
ROMA (%)	7 (5, 11)	31 (10, 86)	7 (5, 10)	27 (9, 88)	7 (5, 10)	30 (10, 86)
**ROMA (cutoff: 10%)**						
Low	1,208 (72%)	114 (25%)	539 (75%)	53 (27%)	1,747 (73%)	167 (26%)
High	463 (28%)	344 (75%)	179 (25%)	142 (73%)	642 (27%)	486 (74%)
**ROMA (cutoff: 15%)**						
Low	1,449 (87%)	170 (37%)	649 (90%)	76 (39%)	2,098 (88%)	246 (38%)
High	222 (13%)	288 (63%)	69 (9.6%)	119 (61%)	291 (12%)	407 (62%)
**ROMA (cutoff: 12.5/14.4%** ^a^ **)**						
Low	1,398 (84%)	154 (34%)	619 (86%)	65 (33%)	2,017 (84%)	219 (34%)
High	273 (16%)	304 (66%)	99 (14%)	130 (67%)	372 (16%)	434 (66%)
**ROMA (cutoff: 13.1/27.7%** ^a^ **)**						
Low	1,507 (90%)	196 (43%)	658 (92%)	83 (43%)	2,165 (91%)	279 (43%)
High	164 (9.8%)	262 (57%)	60 (8.4%)	112 (57%)	224 (9.4%)	374 (57%)
CPH-I (%)	1 (1, 3)	11 (2, 76)	1 (1, 2)	11 (2, 83)	1 (1, 3)	11 (2, 79)
**CPH-I (cutoff: 1%)**						
Low	672 (40%)	52 (11%)	296 (41%)	27 (14%)	968 (41%)	79 (12%)
High	999 (60%)	406 (89%)	422 (59%)	168 (86%)	1,421 (59%)	574 (88%)
**CPH-I (cutoff: 3%)**						
Low	1,300 (78%)	127 (28%)	578 (81%)	61 (31%)	1,878 (79%)	188 (29%)
High	371 (22%)	331 (72%)	140 (19%)	134 (69%)	511 (21%)	465 (71%)
**CPH-I (cutoff: 5%)**						
Low	1,467 (88%)	156 (34%)	649 (90%)	73 (37%)	2,116 (89%)	229 (35%)
High	204 (12%)	302 (66%)	69 (9.6%)	122 (63%)	273 (11%)	424 (65%)
**CPH-I (cutoff: 7%)**						
Low	1,540 (92%)	189 (41%)	667 (93%)	86 (44%)	2,207 (92%)	275 (42%)
High	131 (7.8%)	269 (59%)	51 (7.1%)	109 (56%)	182 (7.6%)	378 (58%)

Statistics presented: median (IQR), *n* (%); EOC, epithelial ovarian cancer.

^a^ Premenopausal/postmenopausal women.

### Optimal cutoff value

**Figure 2:**
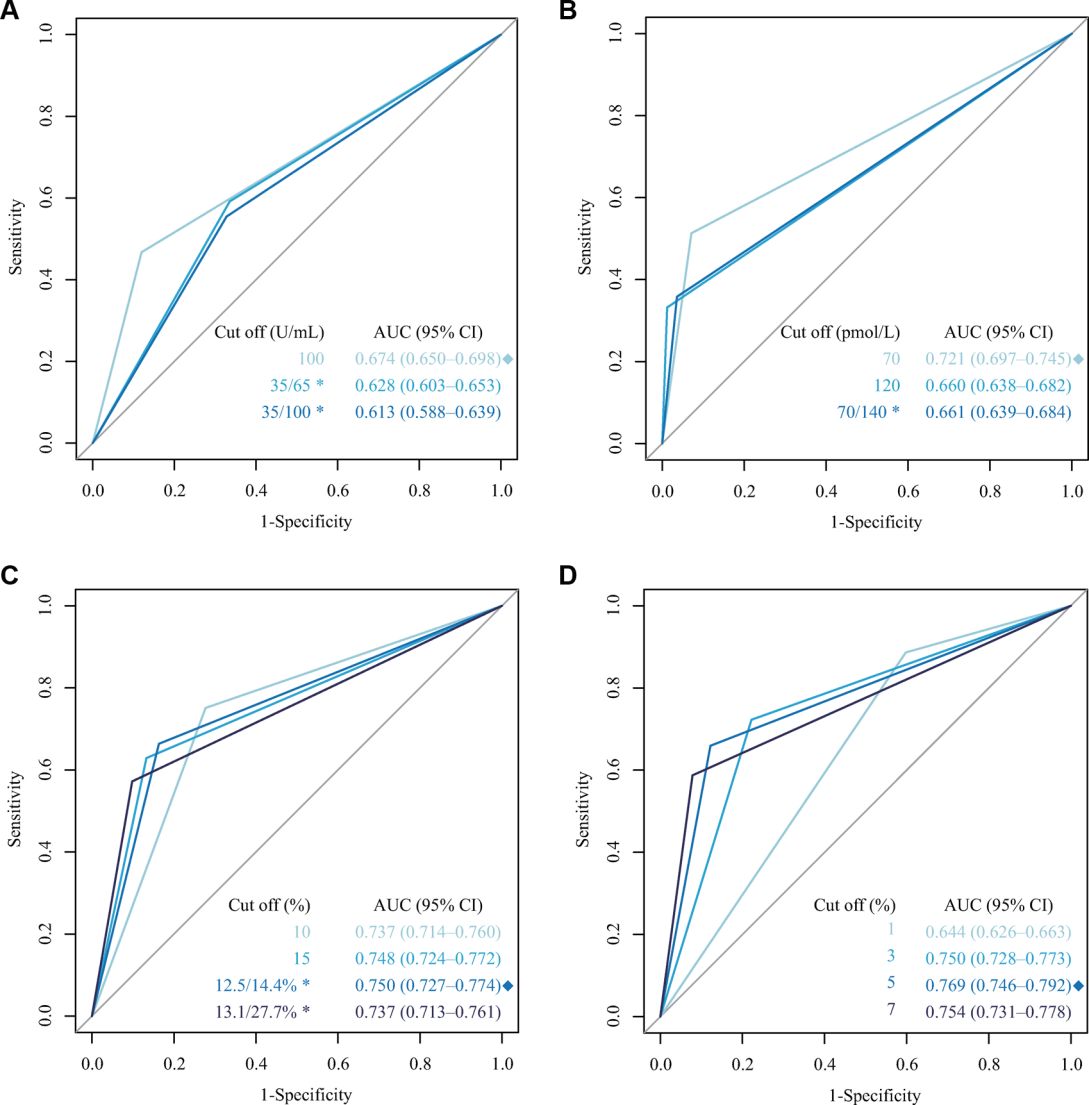
Receiver operating characteristic (ROC) curve for different cutoffs of CA125, HE4, ROMA and CPH-I. (**A**) ROC curve of different cutoffs for CA125; (**B**) ROC curve of different cutoffs for HE4; (**C**) ROC curve of different cutoffs for ROMA; (**D**) ROC curve of different cutoffs for CPH-I. *: Premenopausal/postmenopausal women; ◆: The highest AUC.

In the training cohort data, ROC curves were drawn based on the different cutoff values of CA125, HE4, ROMA, and CPH-I, as shown in Figure 2. The result shows that for CA125, HE4, ROMA, and CPH-I, the cutoff value was 100 U/ml, 70 pmol/L, 12.5/14.4 L% (premenopausal/postmenopausal women), and 5%, respectively, and the AUC values were 0.674, 0.721, 0.750 and 0.769, respectively, which were closest to 1.0. The AUCs of ROMA and CPH-I were significantly higher than of CA125 and HE4, indicating that ROMA and CPH-I alone were more accurate than CA125 and HE4 alone in predicting EOC. Optimal cutoff values were selected for ROMA and CPH-I for subsequent EOC risk factor analysis.

### Analysis of EOC risk factors

Table 2 shows a univariate analysis of EOC in a training cohort. Older age, menopausal status, higher birth rate, hypertension, and diabetes have been associated with the risk for EOC. It was also associated with multilocular tumors, solid components, bilateral tumors, larger tumor diameters, and ascites on imaging, and high ROMA and CPH-I tumor markers which were classified by optimal truncation.

**Table 2:** The univariable logistic regression analysis of epithelial ovarian cancer.

Variables	OR	95% CI	*P*-value
**Age (years old)**			
<40	Ref		
40–59	4.91	3.80, 6.38	<0.001*
≥60	10.00	7.26, 13.9	<0.001*
**Postmenopausal**			
No	Ref		
Yes	5.52	4.43, 6.89	<0.001*
**Parity**			
0	Ref		
1	3.47	2.18, 5.53	<0.001*
≥2	3.61	2.64, 5.06	<0.001*
**Hypertension**			
No	Ref		
Yes	2.87	2.23, 3.68	<0.001*
**Diabetes**			
No	Ref		
Yes	1.93	1.44, 2.57	<0.001*
**Multilocular tumor**			
No	Ref		
Yes	5.20	4.17, 6.50	<0.001*
**Contains solid components**			
No	Ref		
Yes	7.66	6.12, 9.63	<0.001*
**Bilateral tumor**			
No	Ref		
Yes	2.45	1.88, 3.18	<0.001*
**Largest diameter (cm)**			
<5	Ref		
5–14	1.71	1.24, 2.40	0.001*
≥15	6.65	4.67, 9.61	<0.001*
**Ascites**			
No	Ref		
Yes	7.10	5.32, 9.52	<0.001*
**ROMA (cutoff: 12.5/14.4%** ^a^ **)**			
Low	Ref		
High	10.1	8.02, 12.8	<0.001*
**CPH-I (cutoff: 5%)**			
Low	Ref		
High	13.9	10.9, 17.8	<0.001*

OR, odds ratio; CI, confidence interval; Ref, reference.

^a^ Premenopausal/postmenopausal women.

* *P* < 0.05.

Multivariate logistic regression analysis was conducted on the meaningful variables in the univariate logistic regression analyses. Since CA125 and HE4 were included both in the ROMA and CPH-I formulas, multifactor logistic regression analysis was performed for ROMA and CPH-I using clinical and imaging data, respectively. Because the ROMA calculation included menopause, menopausal status was no longer included in ROMA's multivariate analysis. Similarly, age was not included in the multivariate analysis of CPH-I. Multivariate analysis results are shown in Table 3. Multivariate logistic regression analysis involving ROMA showed that older age, imaging findings of multilocular tumors, solid components, bilateral tumors, larger tumor diameter, ascites, and high-risk ROMA were associated with a higher risk of EOC. The results of CPH-I's multivariate analyses revealed that menopausal status, imaging findings of multilocular tumors, solid components, bilateral tumors, larger tumor diameter, ascites, and high-risk CPH-I were associated with EOC.

**Table 3:** The multivariate logistic regression analysis of epithelial ovarian cancer.

Variables	Contains ROMA			Contains CPH-I		
	OR	95% CI	*P*-value	OR	95% CI	*P*-value
**Age (years old)**						
<40	Ref			–	–	–
40–59	1.88	1.29, 2.75	<0.001*	–	–	–
≥60	2.45	1.40, 4.30	0.002*	–	–	–
**Postmenopausal**						
No	–	–	–	Ref		
Yes	–	–	–	2.38	1.66, 3.42	<0.001*
**Parity**						
0	Ref			Ref		
1	1.31	0.84, 2.07	0.236	1.36	0.89, 2.12	0.168
≥2	0.98	0.50, 1.90	0.956	0.9	0.46, 1.74	0.753
**Hypertension**						
No	Ref			Ref		
Yes	1.15	0.76, 1.74	0.499	1.18	0.78, 1.78	0.425
**Diabetes**						
No	Ref			Ref		
Yes	0.96	0.62, 1.46	0.852	0.96	0.61, 1.51	0.866
**Multilocular tumor**						
No	Ref			Ref		
Yes	3.4	2.57, 4.52	<0.001*	3.7	2.75, 4.99	<0.001*
**Contains solid components**						
No	Ref			Ref		
Yes	5.12	3.85, 6.82	<0.001*	5.83	4.31, 7.94	<0.001*
**Bilateral tumor**						
No	Ref			Ref		
Yes	2.49	1.73, 3.59	<0.001*	2.67	1.81, 3.92	<0.001*
**Largest diameter (cm)**						
<5	Ref			Ref		
5–14	1.5	1.00, 2.30	0.055	1.38	0.90, 2.17	0.148
≥15	4.28	2.69, 6.91	<0.001*	4.05	2.47, 6.73	<0.001*
**Ascites**						
No	Ref			Ref		
Yes	4.52	3.06, 6.70	<0.001*	4.87	3.24, 7.35	<0.001*
**ROMA (cutoff: 12.5/14.4%** ^a^ **)**						
Low	Ref			–	–	–
High	4.92	3.63, 6.68	<0.001*	–	–	–
**CPH-I (cutoff: 5%)**						
Low	–	–	–	Ref		
High	–	–	–	9.21	6.75, 12.7	<0.001*

OR, odds ratio; CI, confidence interval; Ref, reference.

^a^ premenopausal/postmenopausal women.

* *P* < 0.05.

### Construction and evaluation of nomograms

**Figure 3:**
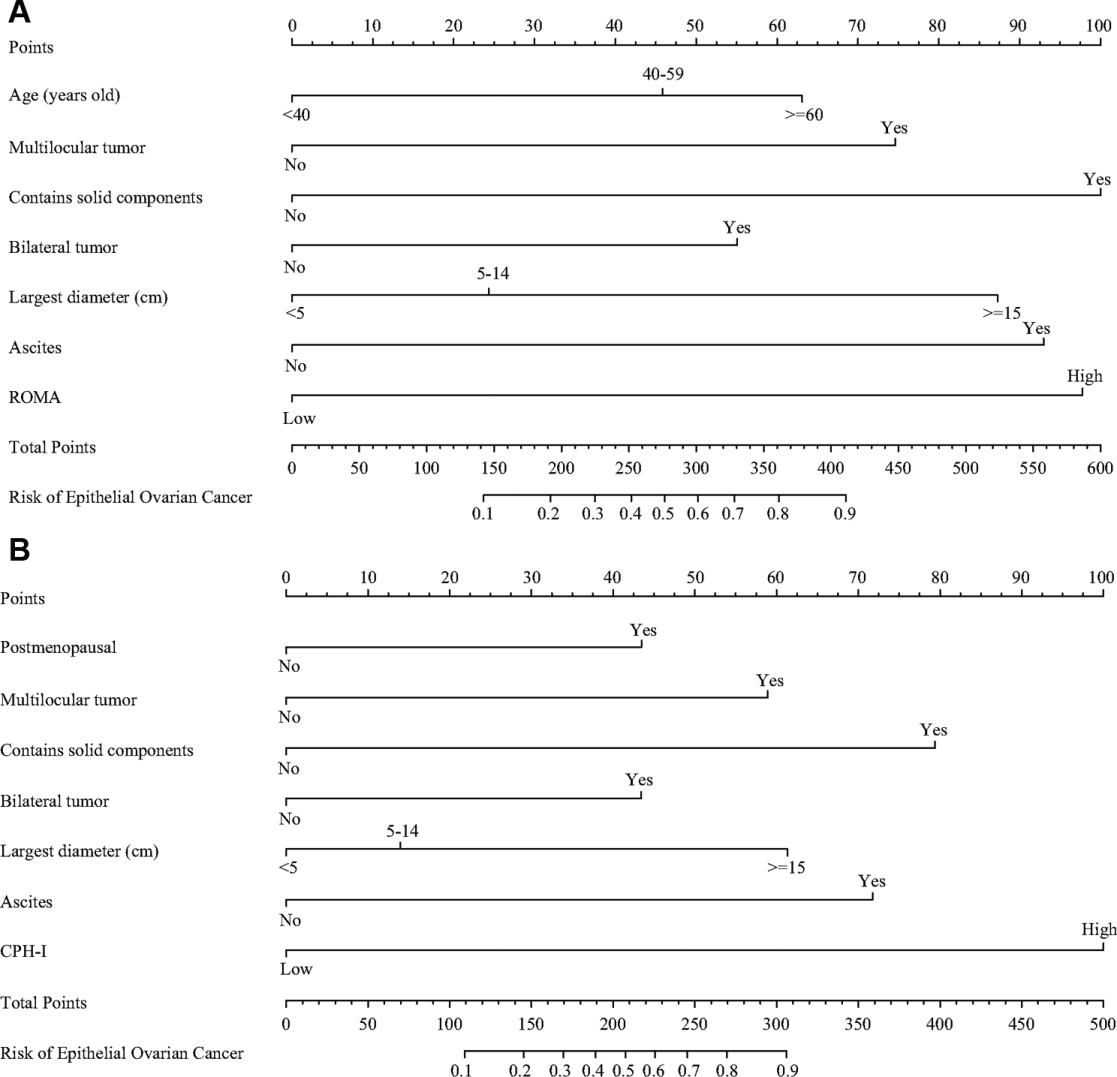
Nomograms of epithelial ovarian cancer. (**A**) Nomograms with ROMA; (**B**) nomograms with CPH-I.

**Figure 4:**
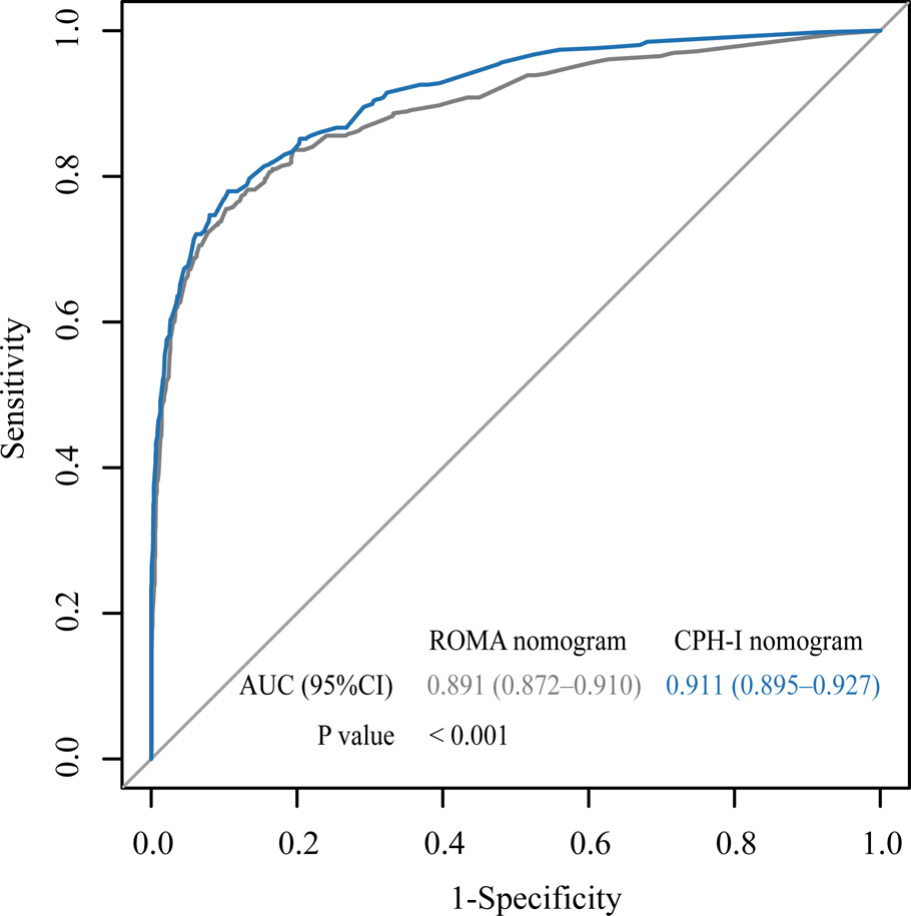
Receiver operating characteristic (ROC) curve for nomograms with ROMA or CPH-I. AUC, area under the curve.

**Figure 5:**
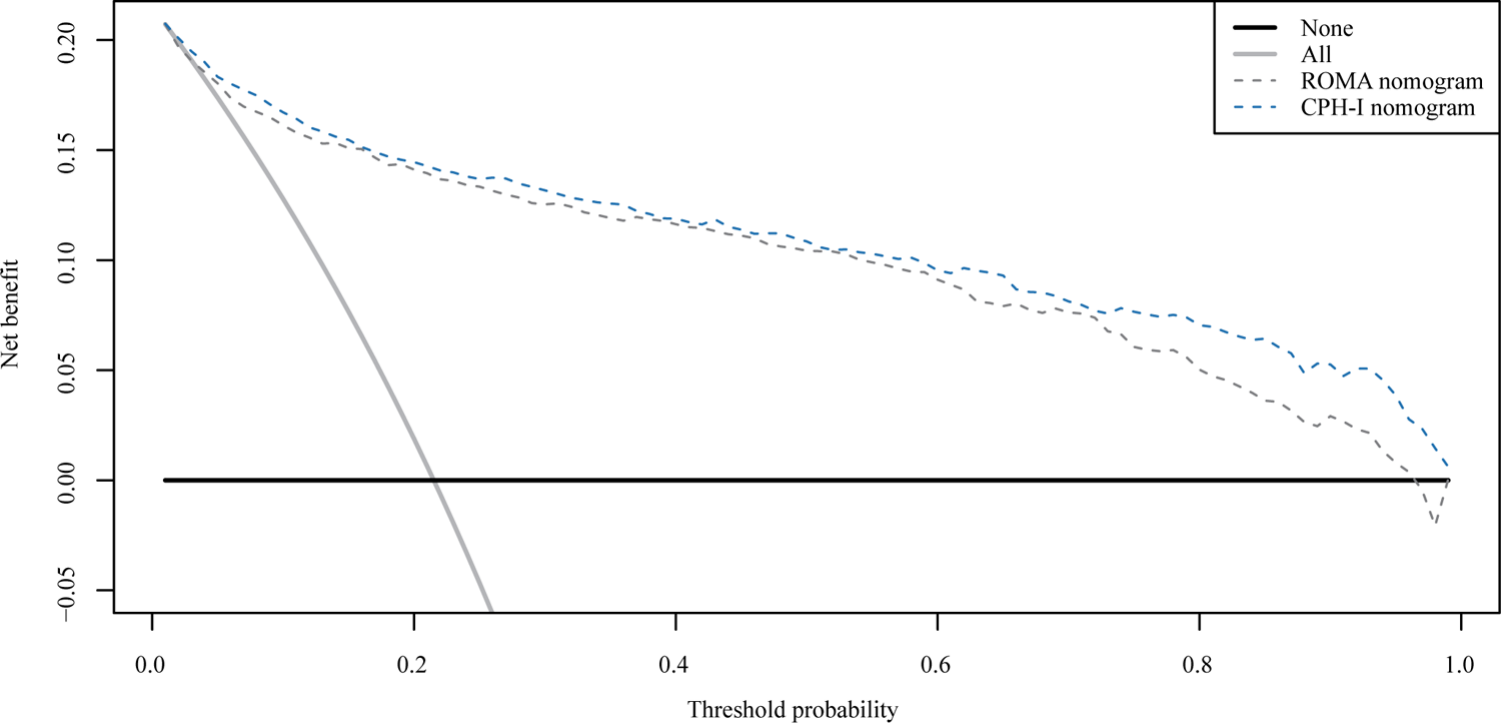
Decision curve analysis (DCA) curve for nomograms with ROMA or CPH-I.

**Figure 6:**
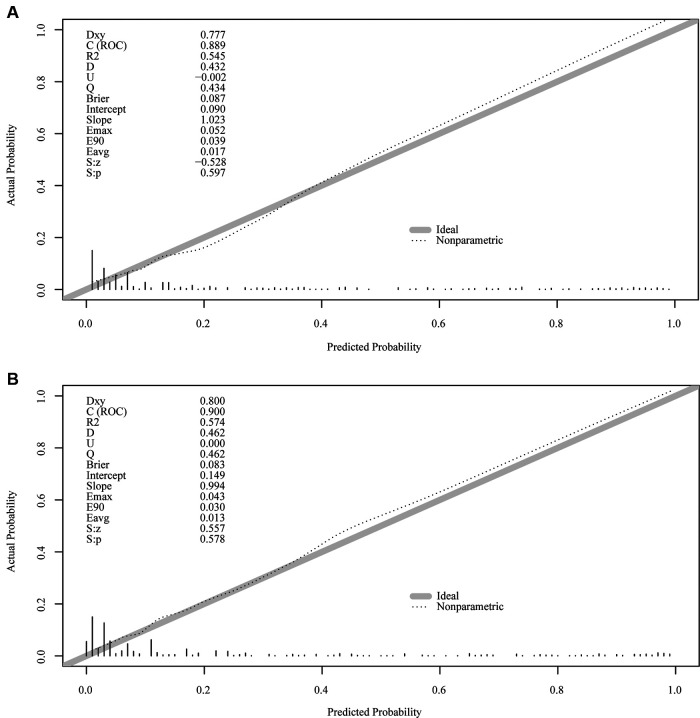
External verification plots of ROMA and CPH-I nomogram verification curves. (**A**) Nomograms with ROMA; (**B**) nomograms with CPH-I.

The important variables for multivariate logistics regression with ROMA included age, radiographically showing multilocular tumors, solid components, bilateral tumor, larger tumor diameter, ascites, and higher ROMA. The important variables for multivariate logistics regression with CPH-I included menopausal status, radiographically showing multilocular tumors, solid components, bilateral tumor, larger tumor diameter, ascites, and higher CPH-I. Nomograms were constructed with ROMA and CPH-I for EOC, using the respective variables for each marker (Figure 3). ROC curves were used to evaluate the accuracy of the nomogram containing ROMA and CPH-I (Figure 4). The AUC of nomograms containing ROMA and CPH-I were 0.8914 and 0.9114, respectively, which were better than the prediction accuracies of CA125, HE4, ROMA, and CPH-I alone. The nomogram with CPH-I was significantly better than that with ROMA (*P* < 0.001). In addition, the DCA of the nomogram containing CPH-I appeared more clinically beneficial than the nomogram containing ROMA (Figure 5). The calibration curve showed that the predictive value of the nomogram for the external validation with ROMA and CPH-I in the test cohort was in close approximation with training chort indicating good agreement between them (Figure 6).

## Discussion

Early ovarian cancer is often not accompanied by significant clinical symptoms because of its deep pelvic location and thick abdominal fat. In addition, most women ignore conventional gynecological examinations, resulting in a relatively low detection rate of OC in this population. OC is often accompanied by extensive metastasis, lack of effective treatment, poor prognosis, and high recurrence rate, causing mortality to always rank first among gynecological malignant tumors.

Many international studies have shown that the detection of serum CA125, HE4, and gynecological ultrasound has great clinical value in malignant risk assessment of ovarian neoplasms (34); the levels of CAl25 and HE4 in patients with malignant tumors are higher than in patients with benign or borderline tumors (21, 35–38). However, both have limitations in screening for ovarian cancer. CA125 levels are significantly increased in approximately 85% of serous ovarian cancers, followed by endometrioid carcinoma, low or no expression in clear cell carcinoma, mucinous ovarian carcinoma, and specific non-epithelial tumors (such as sex cord-stromal tumor and germ cell tumor), and are affected by inflammation (38, 39). Serum HE4 dosage is a useful preoperative test for predicting benign and malignant ovarian disease, and it appears to play a promising role in predicting clinical and surgical outcomes (11). HE4 is abnormally elevated in most serous ovarian carcinoma, endometrioid carcinoma, and clear cell carcinoma of the ovary, but low in mucinous ovarian carcinoma and other non-epithelial ovarian malignancies, and HE4 is affected by the patient's age and renal function (36, 37).

At present, there are many risk assessment systems for ovarian neoplasms that have been developed and reported. The earliest assessment model was the risk of malignancy index (RMI) 1, first proposed by Jacobs in 1990, which included CA125, ultrasound score, and menstrual status score (40). Ultrasonic score includes five indicators: with/without solid area, unilateral/bilateral involvement, multiple rooms or not, ascites or not, and with/without metastases. Each item was assigned one point, and the sum of each item was the total score of the ultrasound. Meanwhile, the longest diameter line of the mass was measured, and postmenopausal status was rated 3 or 4. When the threshold value of RMI1 is 200, relevant studies on postmenopausal people show that RMI1 is 80%–84% sensitive and 87.7%–89% specific (41). In 1996, Tingulstad adjusted ultrasound score and menopause status based on RMI1 and developed RMI2 (42); RMI3 (43) was developed in 1999. RMI was not widely verified in the past 10 years until Yamamoto (44) proposed RMI4 based on RMI2 by adding the maximum diameter of the lump. Liang (45) reported that RMI4 had a sensitivity of 84.9% and a specificity of 93.7%. Timmerman (46) normalized the terms of morphological characteristics of ovarian tumors in 2008 and proposed the IOTA-simple ultrasound rule based on a large-sample retrospective clinical study. Relevant studies have shown that IOTA has a sensitivity of 93%–99% and specificity of 6%–92% for ovarian neoplasms (47, 48). In 2016, Yanaranop (49) proposed ovarian cancer predictive score (R-OPS) for 260 patients combined with their CA125 and HE4 levels, ultrasound score, and menopausal status. The team verified 266 patients and obtained good diagnostic efficacy.

The most widely used and validated clinical indicators are the ROMA and CPH-I. These two indexes are based on serum CA125 and HE4 levels and age or menopausal status but lack imaging indicators. Moore (6) found that when the specificity of RMI and ROMA was set at 75%, ROMA had a sensitivity of 94.6% for predicting ovarian malignancy, whereas RMI had a sensitivity of 84.6%, indicating that ROMA had a better diagnostic performance than RMI. There are many problems with ROMA validation studies, with identifying the menopausal status being the main one. There is no consensus on the definition of menopause in patients who have had their last menstruation or have had a previous hysterectomy or are 50 years old or older, or based on amenorrhea time and the combined determination of FSH and estradiol (50). Clinically, the former is preferred over the latter. Second, the population of patients with ovarian malignancies includes EOC patients and non-EOC patients, such as germ cells. However, the ROMA index was initially established with a preference for diagnosing epithelial ovarian malignancies. The CPH-I index was based on patient age, with a specificity of 88.4% and sensitivity of 82% in women of different regions and ethnicities, and a specificity of 95.2% and a sensitivity of 75.7% in the Asian population (25). The overall diagnostic efficacy of CPH-I was similar to ROMA, with an AUC of 0.951 and 0.953, respectively. However, the different analytical methods used for CA125 and HE4 may distort the diagnosis.

Based on ROMA and CPH-I, this study included ultrasound features contained in the RMI and IOTA and scored them to establish a nomogram for EOC diagnosis. Compared with ROMA or CPH-I alone, these nomograms had higher AUC of 0.8914 and 0.9114, respectively, and better prediction accuracy than CA125 and HE4 alone. DCA of the nomogram containing CPH-I showed better clinical benefits than the nomogram containing ROMA. An efficient malignant risk assessment system can initially shunt ovarian tumors and make correct judgments as soon as possible under nonspecific symptoms and signs, which can guide clinicians in conducting preoperative evaluation and facilitate the next treatment plan. For patients with low CPH-I, evidence-based clinical observation may be selected to avoid laparoscopic exploration, or for patients with high CPH-I but no specific malignant tumor clinical symptoms, early exploration or biopsy may be warned to improve future survival. Of course, some cutting-edge detection methods, such as extracellular vesicles and peripheral blood RNA, can also be referred to improve the level of early diagnosis and treatment (51).

The study still has the following shortcomings. (1) The case data included in this study are still relatively small, which may be biased and cannot fully represent the overall level of nomograms. (2) Only EOC was collected in the study without non-epithelial or other pathological types of tumors. Therefore, the diagnostic analysis of various factors in OC and borderline ovarian tumors should be further explored. (3) The risk cutoff values for ROMA or CPH-I in this study slightly differed from those previously reported. This may be related to the definition of menopausal status, inclusion criteria, ethnicity, differences in ultrasound interpretation, and sample size. (4) CA125 combined with HE4 was not analyzed as a separate set of data, although its results were not necessarily superior to those of CHP-I and ROMA. (5) Furthermore, the data analysis was not staged, so it was impossible to verify the predictive efficacy of the nomogram in different stages of EOC. 6. In the sample of this study, there was a mismatch in the number of pre—and postmenopausal cases, which would have affected the predictability of ROMA and its nomogram. In the future, the sample size should be expanded to include different populations in different regions.

## Conclusion

This study provides a theoretical basis for evaluating the value of predictive models for ovarian masses and a new direction and new idea for the early diagnosis of patients with EOC. CPH-I and ROMA have higher diagnostic values in the preoperative diagnosis of EOC than other single tumor markers such as CA125 or HE4. A nomogram based on CPH-I and ROMA with clinical and ultrasonic indicators had a better diagnostic value, and the CPH-I nomogram had the highest diagnostic efficacy.

## Data Availability

The raw data supporting the conclusions of this article will be made available by the authors, without undue reservation.
